# Potential role of m6A RNA methylation regulators in osteosarcoma and its clinical prognostic value

**DOI:** 10.1186/s13018-021-02422-5

**Published:** 2021-05-05

**Authors:** Hua Liu, Guangzhen Qin, Yanan Ji, Xiaojian Wang, Hailin Bao, Xiaojun Guan, Aichun Wei, Zhigang Cai

**Affiliations:** 1 Department of Orthopedics, Haian Hospital of Traditional Chinese Medicine, 55 Ninghai Middle Road, Haian, Nantong, Jiangsu Province 226600 People’s Republic of China; 2 Key Laboratory of Neuroregeneration of Jiangsu and Ministry of Education, Nantong University, Nantong, Jiangsu Province 226001 People’s Republic of China; 3 Clinical Laboratory, Haian Hospital of Traditional Chinese Medicine, Haian, Nantong, Jiangsu Province 226600 People’s Republic of China

**Keywords:** Osteosarcoma, m6A, m6A regulators, Prognosis, TCGA

## Abstract

**Background:**

Osteosarcoma is a disease with high mortality in children and adolescents, and metastasis is one of the important clinical features of osteosarcoma. *N*6-Methyladenosine (m6A) is the most abundant methylation modification in mRNA, which is regulated by m6A regulators. It is reported that it is related to the occurrence and development of tumors. However, the mechanism of its action in osteosarcoma is rarely known. The purpose of this study was to identify the potential role of m6A regulatory factor in osteosarcoma and its clinical prognostic value.

**Methods:**

Here, we used The Cancer Genome Atlas (TCGA) to comprehensively analyze the relationship between m6A regulatory factors and osteosarcoma (metastasis group and non-metastasis group). We analyzed their survival relationship and analyzed all the m6A regulatory factors in TCGA tumor data set by using the univariate Cox proportional hazard regression model. Finally, we selected two survival-related methylation regulators (FTO and IGF2BP2) as risk gene signature.

**Results:**

According to the median risk, patients were divided into low-risk group and high-risk group. Multivariate Cox regression analysis showed that these two risk genes were considered to be the key factors independently predicting the prognosis of patients with osteosarcoma. In addition, we verified their characteristics with gene expression omnibus (GEO) DataSets and confirmed that they are related to tumor and immune-related signaling pathways through gene set enrichment analysis (GESA) and immune infiltration analysis.

**Conclusions:**

In conclusion, m6A regulators might play an important role in the metastasis of osteosarcoma and have potential important value for the prognosis and treatment strategy of osteosarcoma patients.

## Introduction

Osteosarcoma (OS) is a malignant bone tumor, which is common in children and adolescents [1]. The incidence rate of osteosarcoma is increasing year by year, accounting for 35% of primary bone malignancies. However, the survival rate of patients with osteosarcoma is still low, especially for patients with metastatic osteosarcoma [2, 3]. Once the patients with osteosarcoma have tumor metastasis, the disease is often in the late stage, with poor prognosis and high mortality [4]. So far, the potential molecular mechanism of the occurrence and development of osteosarcoma is still unclear, which hinders the development of its treatment strategy. Therefore, it is an urgent task to provide new therapeutic targets for osteosarcoma.

*N*6-Methyladenosine (m6A) is the most abundant methylation modification of mRNA, which mainly exists in the CDS region and 3′UTR region of mRNA, affecting the stability, translation efficiency, alternative splicing, and localization of mRNA [5]. In addition, non-coding RNAs such as long noncoding RNA and miRNA also have m6A modification sites. M6A modification on mRNA, similar to DNA and protein modification, can be reversibly and dynamically regulated by methyltransferases (writers), m6A-binding proteins (readers), and demethylases (erasers) [6]. Methyltransferase is an important class of catalytic enzymes, which can cause the m6A methylation in mRNA, mainly including METTL3, METTL14, METTL16, WTAP, VIRMA, RBM15, and ZC3H13 [7–10]. Readers are specific RNA binding proteins required by the mRNA modified by m6A to perform specific biological functions, including IGF2BPs, YTHDFs, YTHDCs, FMR1, and HNRNPs [11–13]. They can recognize the information of RNA methylation and participate in the translation and degradation of downstream mRNA. Demethylase, including FTO and ALKBH5, erases m6A modification and regulates the metabolization of mRNAs [14, 15].

In recent years, it has been found that m6A is involved in the pathogenesis and development of tumors. For example, in bladder cancer, the m6A regulator METTL3 promotes the progression of bladder cancer through the AFF4/NF-kB/MYC signaling network [16]. In chemical carcinogenesis, METTL3-m6A-CDCP1 axis has synergistic effect with chemical carcinogens to promote the malignant transformation of urothelial cells and occurrence of bladder cancer in vitro and in vivo [17]. In addition, METTL14 plays an important role in EBV-related tumorigenesis by interacting with the potential oncoprotein EBNA3C encoded by EBV [18]. Circe7 is modified by m6A in cytoplasm and translated into E7 (a carcinogenic protein), which provides new insights into how HPV regulates infection and tumorigenesis [18, 19]. However, up to now, the role of m6A regulators in osteosarcoma remains unclear. In this study, we used the osteosarcoma data from The Cancer Genome Atlas (TCGA) to systematically analyze 25 widely reported m6A regulators. We demonstrated the role of m6A regulators in patients with osteosarcoma through bioinformatics analysis. We obtained risk genes through univariate and multivariate regression analysis and explored their clinical prognostic value. More importantly, we have verified their characteristics with gene expression omnibus (GEO) DataSets and confirmed their significance as a new biomarker for patients with osteosarcoma.

## Materials and methods

### Data acquisition

The TARGET-OS gene expression (RNA-Seq) and corresponding clinical data of 88 osteosarcoma patients were downloaded from the Cancer Genome Atlas (TCGA) repository (https://gdc-portal.nci.nih.gov/). GEO dataset (GSE21257) were obtained from the Gene Expression Omnibus (GEO) database (https://www.ncbi.nlm.nih.gov/geo/). One dataset of OS samples was downloaded from TCGA (Project ID: TARGET-OS), with metastases (*n*=22), without metastases (*n*=66). GSE21257 were obtained from the Gene Expression Omnibus (GEO) with metastases within 5 years (*n*=34) and without metastases within 5 years (*n*=19). Principal component analysis (PCA) was used to observe the gene expression consistency within groups [20].

### m6A methylation regulators

In this study, 25 m6A methylation regulators that have been reported were collected. We then systematically compared the expression of these m6A methylation regulators with clinicopathologic parameters in patients with osteosarcoma.

### Bioinformatics analysis

To further explore the role of m6A methylation regulators in osteosarcoma patients, we used boxplot analysis to analyze the expression level of m6A methylation regulators in both metastatic and non-metastatic osteosarcoma samples. The R package Deseq2 and R package edgeR [21, 22] were used to analyze the differences of each gene in the preclassified samples. GO analysis and KEGG pathway analysis were performed using *P* value<0.05 and fold change >2 as the criteria for screening differentially expressed genes (DEGs). Complete distance method was used to perform cluster analysis on the samples according to the expression of m6A methylation regulators in TCGA and draw a heat map. GSEA was used to analyze the functions of the two survival-related regulators, and the samples were divided into two groups according to the expression level of the regulators. Then the log2 differential multiple of the genes detected in the data in the two groups was analyzed (high versus low), and the genes were ranked according to the log2 differential multiple (from large to small). KEGG gene sets (v6. 0) was used (dataset obtained from https://www.gsea-msigdb.org/gsea/msigdb). GSEA was performed by fgsea (v 1.12.0) package. Pathways with p.adj<0.05 and NES absolute≥1 in the screening results were considered to be enrichment pathways [23]. The GEO dataset (GSE21257) was used to verify the characteristics of survivor-related regulators, and principal component analysis (PCA) was used to observe the sample distribution. Finally, in order to analyze the difference of the proportion of immune cells in IGF2BP2 high and low expression samples, the TCGA dataset was analyzed by Timer (tumor immune estimation resource; https://cistrome.shinyapps.io/timer) [24] and Cibersort (https://cibersort.stanford.edu/) [25], respectively. Timer was used to analyze the tumor-infiltrating immune cells and the correlations between the infiltrating level of different subsets of immune cells and IGF2BP2 high and low expression samples. Cibersort was used for estimating the abundance of different immune cell types in tumor microenvironment by FPKM data [26].

### Construction of gene signature

All the m6A methylation regulators in the TCGA tumor dataset were analyzed using univariate Cox proportional risk regression model, and two methylation regulators associated with survival were screened out. Then, multivariate regression analysis was conducted on the two m6A methylation regulators. Univariate and multivariate Cox proportional hazards regression analysis was done by “coxph” function with default parameter of R “survival” package, version 3.2-7 [27]. Then, choose a model by AIC in a Stepwise Algorithm [28], the mode of stepwise search is “both”, and a risk formula was constructed according to Cox regression results.

$$ \mathrm{Risk}\ \mathrm{formula}=\hbox{-} 1.066\ast \mathrm{FTO}+0.271\ast \mathrm{IGF}2\mathrm{BP}1 $$

The risk coefficient of each patient in TCGA tumor data was calculated according to the formula, and patients were classified into low-risk group and high-risk group according to the median risk. Those below the median risk were considered low risk, while those above the median risk were considered high risk. Survival curves were drawn to compare the survival status of the two groups.

### Statistical analysis

Univariate and multivariate regression analyses were performed for potential prognostic factors such as age, sex, and risk status. The clinical data of the OS samples provided by TARGET-OS project includes age, gender, race, overall survival time and vital status, metastasis or not, and primary tumor site. Tumor grade, subtype, and size were not provided. The *P* value of enrichment degree was calculated by hypergeometric distribution test for GO enrichment analysis and KEGG enrichment analysis, and *P* value<0.05 was considered as significant.

## Results

### Expression level of m6A methylation regulators in osteosarcoma samples

**Fig. 1:**
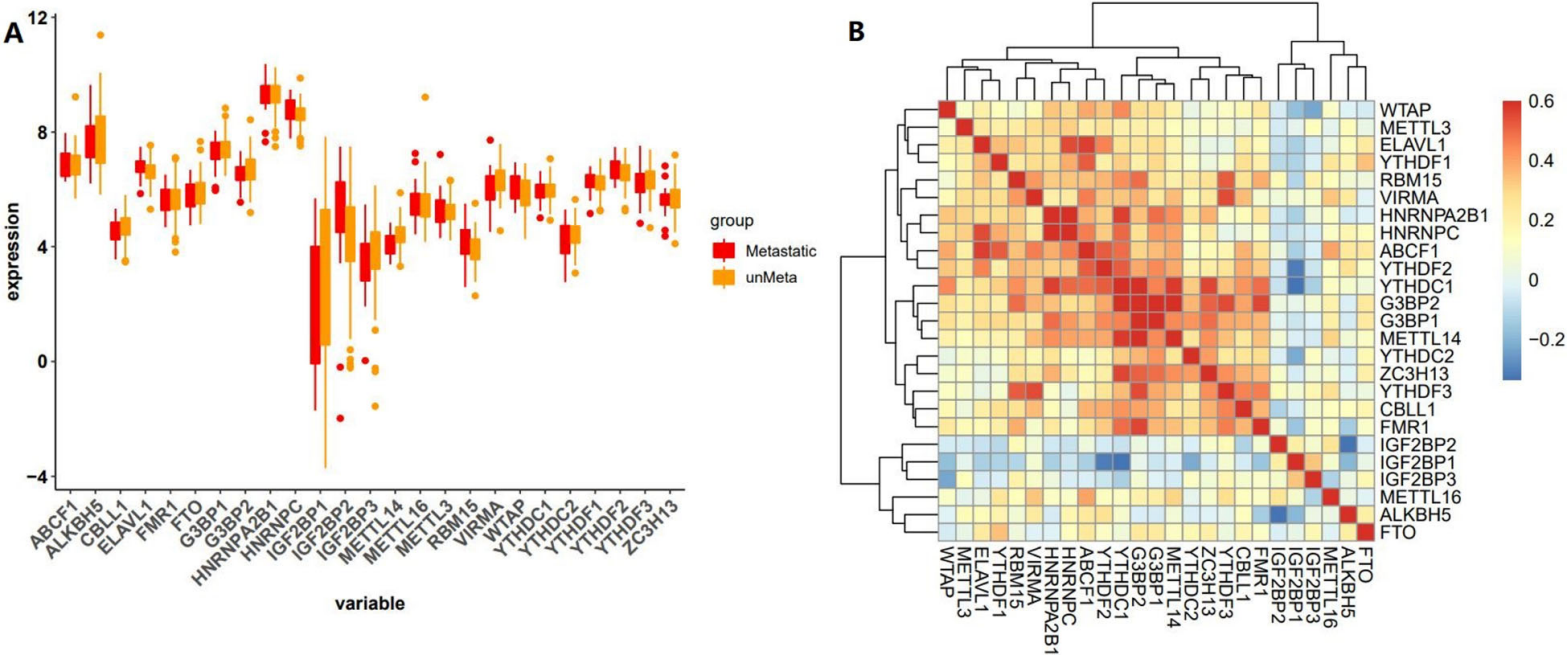
The expression level and correlation of m6A methylation regulators in osteosarcoma samples (metastatic and non-metastatic). **a** Box-plot of all m6A methylation regulators. **b** Correlation diagram of expression of m6A methylation regulators. The higher the correlation, the redder the color, and conversely, the bluer it is

Given the important role of m6A methylation regulators in tumor genesis and progression, we comprehensively analyzed the relationship between all m6A methylation regulators and osteosarcoma using the TCGA dataset. The expression levels of m6A methylation regulators in the metastatic and non-metastatic osteosarcoma samples were presented in boxplot form (Fig. 1a). It is evident from this figure that the expression of most of the m6A methylation regulators is associated with metastasis of osteosarcoma. To better understand the interactions among the m6A methylation regulators, we also analyzed their correlations (Fig. 1b). We speculate that the change in the correlation of m6A methylation regulators may be an intrinsic feature that reflects external differences. The results showed that FTO, ALKBH5 and IGF2BPS had poor correlation with the expression of other m6A methylation regulators. This suggests that these m6A methylation regulators may be significantly related to the metastasis of osteosarcoma.

### Functional annotation of differentially expressed genes by GO analysis and KEGG analysis

**Fig. 2:**
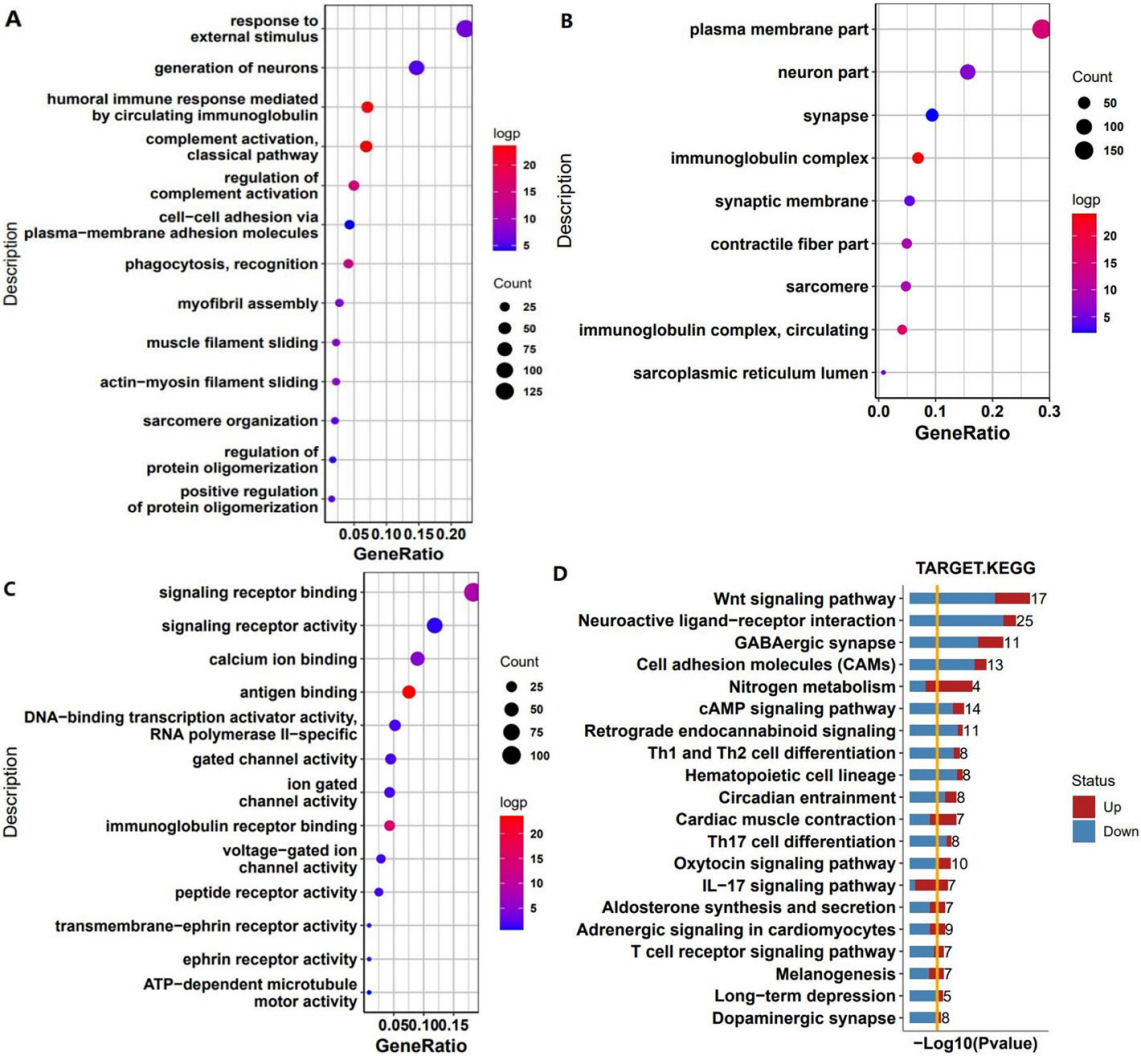
Functional analysis of differentially expressed genes. The differentially expressed genes were analyzed by GO. **a** Biological processes. **b** Cellular components. **c** Molecular functions. Note: The gradient from red to blue indicates that *P* value increases from small to large. The circles go from large to small, representing the number of different genes going from large to small. **d** KEGG enrichment analysis of differentially expressed genes. The top 20 with *P* value<0.05 and DEG ≥3 (number of differences) were selected for plotting. Note: The horizontal axis represents -log10 (*p* value), and the number on the right represents the number of differences

In order to better understand the mechanism between osteosarcoma and 25 m6A methylation regulators, differentially expressed genes were obtained by comparing gene expression between metastatic and non-metastatic osteosarcoma samples. In order to summarize the potential functions of differentially expressed genes, we annotated the function of differentially expressed genes through GO function analysis and KEGG pathway analysis. Firstly, these differentially expressed genes were analyzed by GO. It can be clearly seen from the figure that biological processes are mainly concentrated in the classical pathway of complement activation and humoral immune response mediated by circulating immunoglobulin (Fig. 2a), cell components are concentrated in immunoglobulin complex (Fig. 2b), and molecular functions are concentrated in antigen binding and immunoglobulin receptor binding (Fig. 2c). Then, through KEGG pathway analysis, we found that the upregulated genes were mainly related to nitrogen metabolism, cardiac muscle contraction, and IL-17 signaling pathway, while the downregulated genes were related to Wnt signaling pathway, neuroactive ligand-receptor interaction, GABAergic synapses, dopaminergic synapse, cell adhesion molecules, and T17 cell differentiation (Fig. 2d). Looking at the results of these analyses, we can speculate that the metastasis of osteosarcoma may be closely related to immunity.

### Prognostic value of m6A methylation regulators in osteosarcoma

**Fig. 3:**
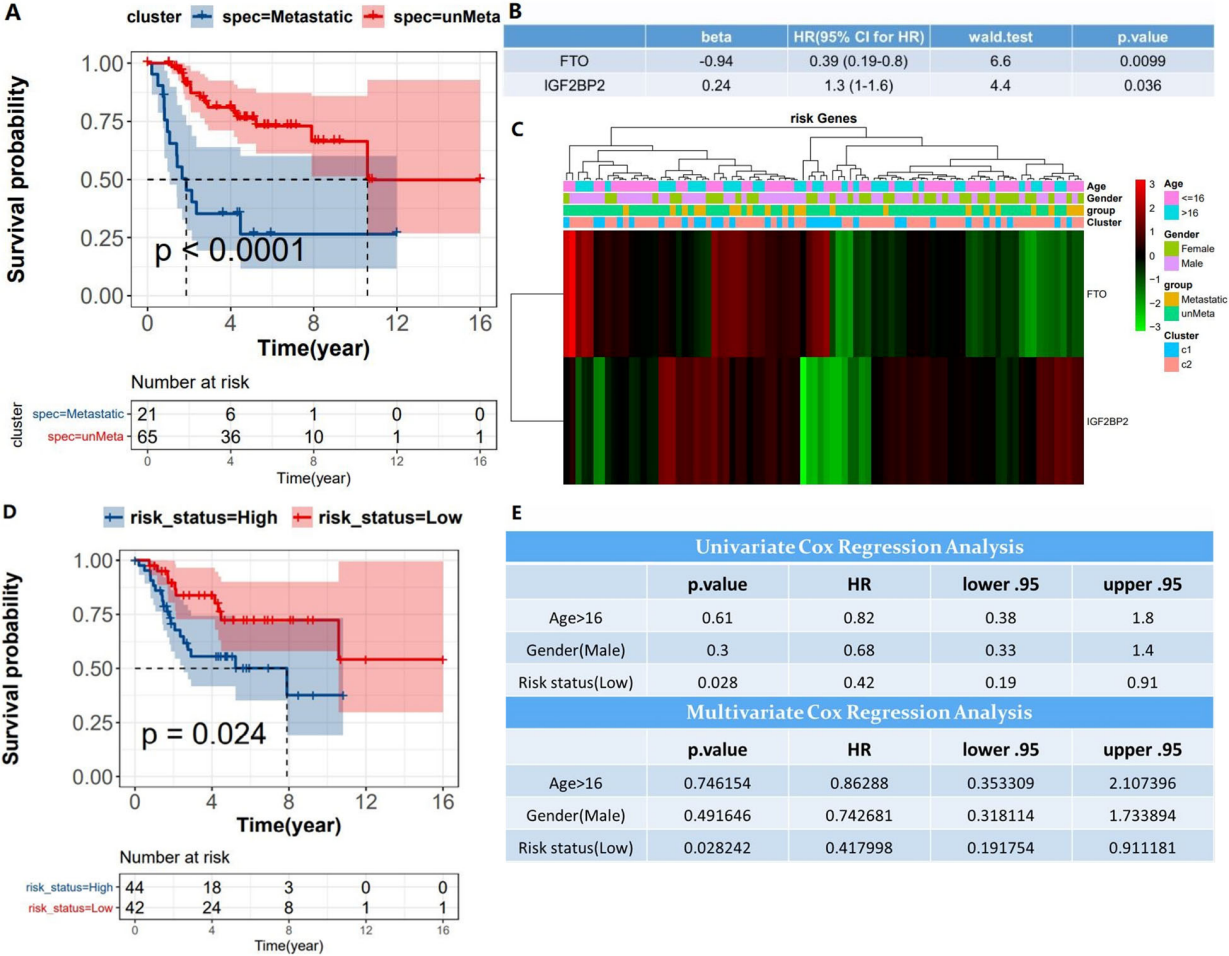
Effect of m6A methylation regulators on prognosis and clinicopathologic features of osteosarcoma. **a** Relationship between metastatic and non-metastatic samples and survival. **b** Univariate Cox proportional risk regression model obtained two survival-related methylation regulators (*P* <0.05). **c** Heat maps showed the expression levels of the two m6A methylation regulators and clinicopathological distribution in both metastatic and non-metastatic groups. **d** Survival analysis of patients in the high-risk and low-risk groups. **e** Survival analysis for age, sex, and risk status was performed using univariate and multivariate regression

To investigate the prognostic value of the m6A methylation regulators, we first analyzed the relationship between the metastatic and non-metastatic groups and survival (Fig. 3a). Interestingly, we found that the metastatic and non-metastatic groups were associated with survival, and survival was worse in the metastatic group than in the non-metastatic group. Next, we used univariate Cox proportional risk regression model to analyze all the m6A methylation regulators in the TCGA tumor dataset. Finally, two methylation regulators related to survival were screened out, among which FTO was a protective gene (HR<1) and IGF2BP2 was a dangerous gene (HR>1) (Fig. 3b). In order to investigate the prognostic value of risk gene signature in the TCGA database, we designed a heat map to display the clinicopathological characteristics distribution and the expression levels of the two m6A methylation regulators in both metastatic and non-metastatic samples (Fig. 3c). In this heat map, we found a strong correlation between risk gene signature and the clinical features of osteosarcoma. We then performed multivariate Cox regression analysis for these two m6A methylation regulators, dividing patients into low-risk group and high-risk group according to median risk, and plotting survival curves (Fig. 3d). As you can see from the graph, low-risk patients fared better than high-risk patients. Finally, univariate and multivariate regression analyses were performed to analyze survival for age, sex, and risk status (Fig. 3e). Risk status was associated with survival in both univariate and multivariate outcomes. The results suggest that risk status may be an independent prognostic factor.

### Validation of the risk signature using data collected from GEO dataset and the exploration of signaling pathways that they involve

**Fig. 4:**
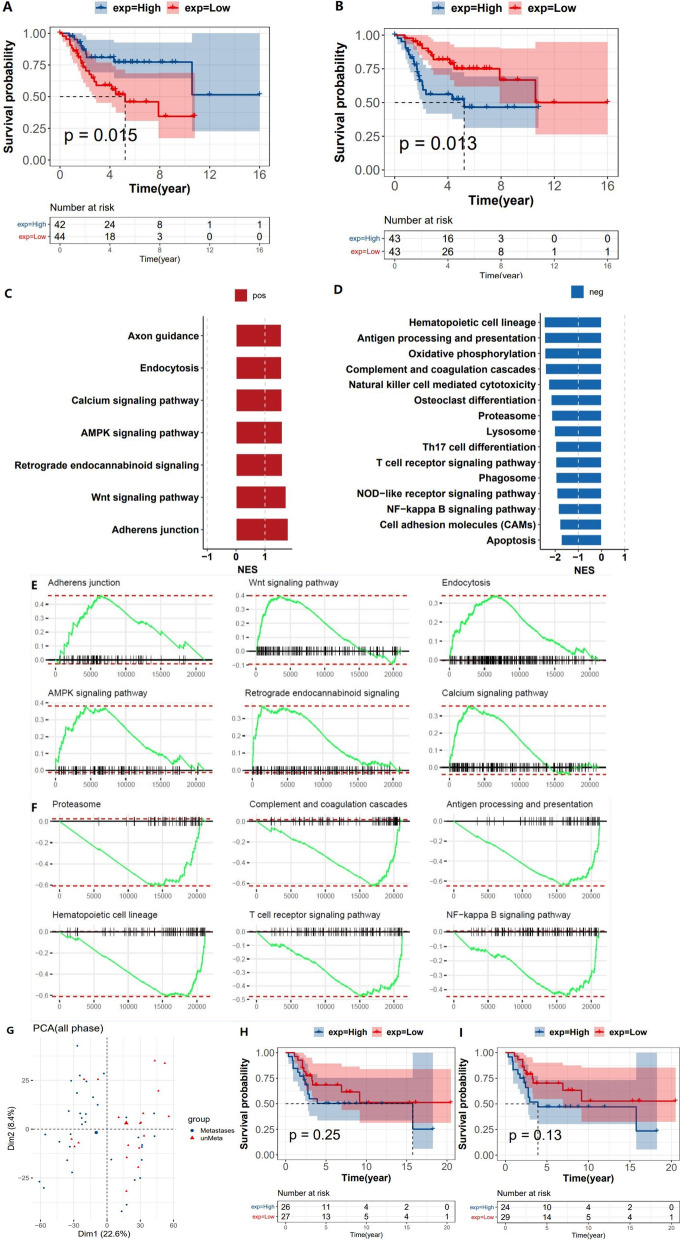
Survival curve analysis, GSEA analysis, and validation in GEO dataset of two survival-related regulatory factors. **a** FTO survival curve. **b** IGF2BP2 survival curve. **c**, **e** GESA analysis of samples with high FTO expression (a total of 7 enriched pathways were screened out for histogram). **d** GESA analysis of IGF2BP2 high expression samples (histogram of the top 15 pathways ranked by P value from small to large by screening the enriched pathways). **e**, **f** Select the top 6 pathway (ranked from small to large by *p* value) to make the distribution curve of enrich score. The vertical axis represents the ES value of this pathway. Positive ES indicates upregulated activity of this pathway, and negative ES indicates downregulated activity of this pathway. **g**–**i** The GEO dataset was used to verify the characteristics of IGF2BP2. **g** PCA analysis of data. **h**, **i** Survival curves of the samples are divided into two categories according to the median value and the mean value

In order to understand the prognostic importance of Gene Signature, we first performed survival curve analysis of these two survival-related regulators (Fig. 4a, b). It can be intuitively seen from the figure that the samples with high FTO expression had good survival, while the samples with high IGF2BP2 expression had poor survival. Through gene-set enrichment analysis (GESA), we found that adhesion junction, Wnt signaling pathway, AMPK signaling pathway, and endocytosis tended to be activated in samples with high FTO expression, and immune-related signaling pathways, oxidative phosphorylation, proteasome, and lysosome tended to be downregulated in samples with high IGF2BP2 expression (Fig. 4c–f). Next, we use the GEO dataset to verify the IGF2BP2 characteristics. This dataset does not have FTO information, so we will not discuss it. We performed PCA analysis of the data (Fig. 4g). It is clear from the PCA plot that there are no outliers. Then, we divided the samples into two categories according to the median value and the mean value and drew survival curves (Fig. 4h, i). From the results, although *P* value was not satisfied, the trend was similar to TCGA, and the results of GSEA were also similar to TCGA. That is, IGF2BP2 may affect patient survival by affecting immune function.

### Immune cell infiltration analysis

**Fig. 5:**
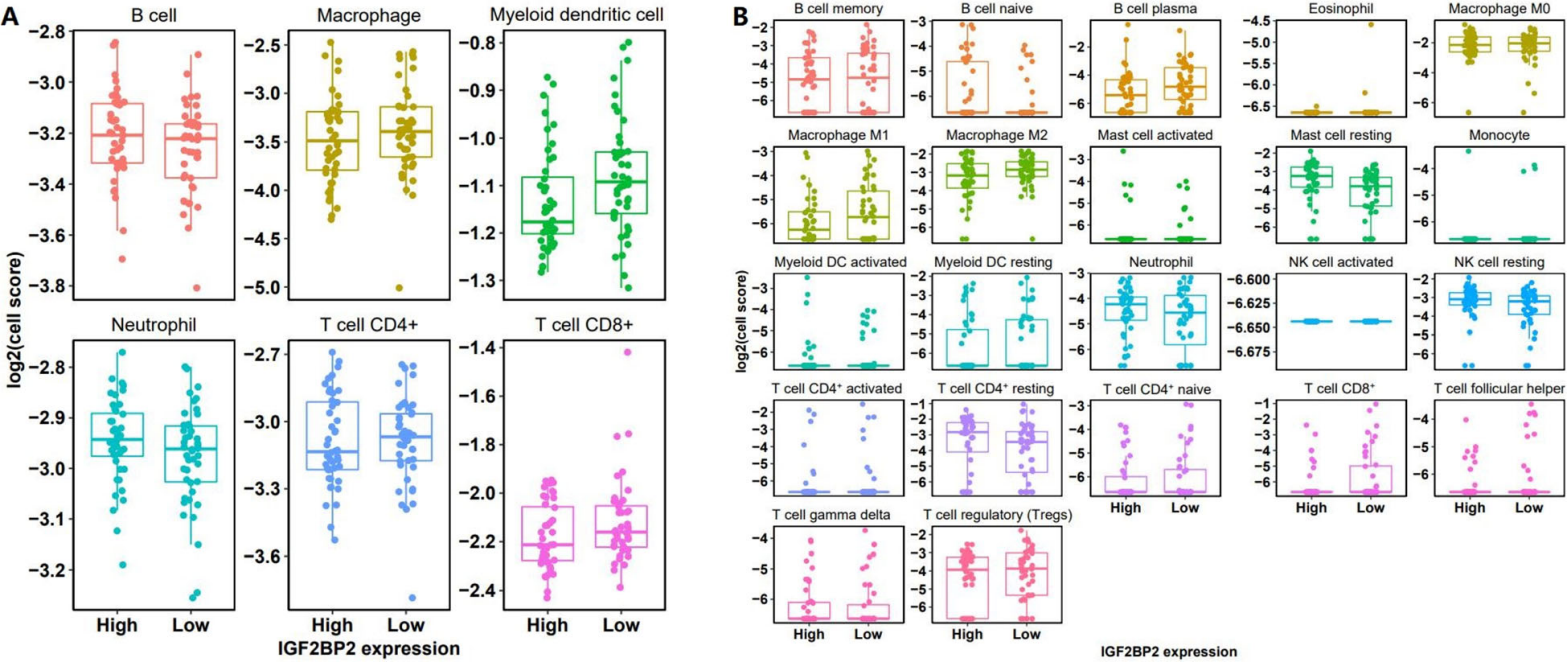
Immune cell infiltration analysis. **a** Timer and **b** Cibersort analysis of differences in the proportion of immune cells in IGF2BP2 high and low expression samples. The higher log2 cell fraction score is, the higher cell abundance is

In order to analyze the difference in the proportion of immune cells in the samples with high and low IGF2BP2 expression, Timer and Cibersort were used to analyze the immune cell infiltration respectively (Fig. 5a, b). Combining with the results of Timer and Cibersort, it is easy to see that the samples with low expression of IGF2BP2 tend to have high abundance of most immune cells (especially Macrophage M1 and M2, T cell CD8^+^, Tregs, DC). B cell naive, mast cell resting, neutrophil, NK cell resting, T cell CD4+ resting, and T cell gamma delta had higher concentrations in the group with high expression of IGF2BP2. These results further suggest that immunity may play an important role in the metastasis of osteosarcoma.

## Discussion

Previous studies have shown that the upregulation or downregulation of m6A methylation regulator is associated with the occurrence of many different tumors, and the same m6A methylation regulator may have different functions in different tumors, such as acute myeloid leukemia, glioblastoma, non-small cell lung cancer, liver cancer, and breast cancer [29–34]**.** Classical epigenetics (DNA or protein modifications) play a key role in the development, progression, and prognosis of osteosarcoma [35, 36]. In this study, we found that the expression of the epigenetic regulator m6A RNA methylation was also closely associated with the severity and prognosis of osteosarcoma.

Here, we first analyzed the expression level of m6A methylation regulators in samples from both metastatic and non-metastatic osteosarcoma groups and their interactions. Then, functional annotation of differential genes was performed by GO functional analysis and KEGG pathway analysis. According to the results, we can conclude that most of the differential genes are closely related to immune function. This provides a direction for our follow-up research. Next, we first analyzed the survival relationship between the metastatic and non-metastatic samples, and we were pleasantly surprised to find that there were significant differences between them, indicating that specific m6A methylation regulators may affect the survival of patients with osteosarcoma. Therefore, we selected two m6A methylation regulators (FTO and IGF2BP2) using univariate Cox proportional risk regression model to explore their prognostic value in osteosarcoma and establish gene signature. The univariate and multivariate regression analyses were used to determine that risk status might be an independent prognostic factor. Finally, we used the GEO dataset and GESA analysis to validate the prognostic value of the two selected m6A methylation regulators and found that IGF2BP2’s influence on survival may be mediated by pathway influence on immunity, which was again confirmed by the immunoinfiltration analysis.

In recent years, FTO has been found to be the first mRNA of m6A demethylase [37]. As an “eraser” of m6A, FTO can remove m6A modification, regulate mRNA stability, and eventually lead to changes in the pathogenesis of various cancers. For example, in glioblastoma stem cells (GSCs), treatment with the FTO inhibitor MA2 increases m6A levels in GSCs, inhibits GSC growth, and prolongs the lifespan of GSC-transplanted mice. The FTO inhibitor MA2 can inhibit GSC-initiated brain tumor development, suggesting that M6A methylation may be a promising target for anti-glioblastoma therapy [30]. In addition, FTO, as an m6A demethylase, has been found to play an important role in carcinogenesis in acute myeloid leukemia (AML). FTO is highly expressed in AML. FTO regulates the expression of target genes such as ASB2 and RARA by decreasing the level of m6A, promotes leukemic oncogene-mediated cell transformation and leukemogenesis, and inhibits the differentiation of AML cells induced by all-trans retinoic acid (ATRA) [38]. However, in osteosarcoma, we found that FTO is a protective gene, and high FTO expression promotes survival in patients with osteosarcoma. Our results are consistent with the following conclusions. Reduced FTO nuclear expression has been reported to be associated with poor prognosis in human hepatocellular carcinoma [39]. FTO expression is downregulated in thyroid cancer tissues, which is associated with lymph node metastasis in thyroid cancer patients [40]. However, FTO also has a role in promoting cancer. For example, FTO plays an important oncogenic role in regulating the proliferation and migration of cervical cancer cells through m6A modification that controls E2F1 and Myc transcription. Studies have shown that FTO is a potential candidate for the treatment of cervical cancer [41]. In addition, USP7 gene knockout or drug suppression (P5091 or P22027) reduced the proliferation rate and colony-forming ability of lung cancer cells, while the inhibitory effect of FTO gene knockout on the growth of lung cancer cells was restored by excessive USP7 expression. These results suggest that m6A demethylase FTO promotes the growth of NSCLC cells by increasing the expression of USP7 [42]. More and more evidence shows that FTO plays different roles in the occurrence and development of different cancers. FTO plays an oncogene role in early acute myeloid leukemia, lung cancer, and cervical cancer and plays a tumor suppressor gene role in hepatocellular carcinoma and thyroid cancer. Our data showed that FTO might play a tumor suppressor gene role in osteosarcoma.

In contrast, IGF2BP2 was found to be a risk gene for osteosarcoma, and high expression of IGF2BP2 inhibited survival in patients with osteosarcoma. There is experimental evidence that IGF2BP2 can promote the metastasis of colorectal cancer through the formation of circNSUN2/IGF2BP2/HMGA2 protein ternary complex in the cytoplasm [43]. IGF2BP2 is frequently overexpressed in pancreatic ductal adenocarcinoma (PDAC) and gallbladder carcinoma (GBC) and is significantly associated with poor prognosis. IGF2BP2 appears to promote tumor progression in PDAC and GBC. Therefore, it may be an interesting prognostic marker and a new target for the treatment of PDAC and advanced GBC [44, 45]. Importantly, IGF2BP2 also acts as an inhibitor in many cancers. Silencing HOTAIR can inhibit the invasion, proliferation, and migration of colon cancer LoVo cells and promote cell apoptosis by inhibiting IGF2BP2 and epithelial mesenchymal transformation [46]. In addition, miR22HG significantly promoted apoptosis and inhibited invasion of cervical cancer cells by targeting IGF2BP2. The long non-coding RNA miR22HG targeting IGF2BP2 as a tumor suppressor for cervical cancer will help us develop potential therapies for cervical cancer [47]. In summary, like FTO, IGF2BP2 also plays different roles in different tumors. We demonstrated that IGF2BP2 was a risk gene for osteosarcoma, and high expression of IGF2BP2 was associated with poor survival in patients with osteosarcoma.

## Conclusions

In this study, we comprehensively analyzed the relationship between the expression of m6A methylation regulators and the occurrence, development, and prognosis of osteosarcoma. Among the m6A methylation regulators, the abnormal expression of FTO and IGF2BP2 was found to be significantly associated with the progression of osteosarcoma and is considered to be the key factors that can independently predict the prognosis of patients with osteosarcoma. The detailed analysis confirmed the important role of IGF2BP2 in the survival of patients with osteosarcoma. In conclusion, our study provides a new marker for evaluating the prognosis of osteosarcoma and provides important evidence for further investigation of the role of m6A methylation regulators in osteosarcoma. However, our study still needs further clinical verification.

## Data Availability

All the data of the manuscript are presented in the paper or additional supporting files. The RNA-Seq and corresponding clinical data were downloaded from the Cancer Genome Atlas repository. GEO dataset (GSE21257) were obtained from the Gene Expression Omnibus database.

## Abbreviations

TCGA: Cancer Genome Atlas
m6A: *N*6-Methyladenosine
GEO: Gene expression omnibus
OS: Osteosarcoma
GESA: Gene set enrichment analysis
PCA: Principal component analysis
DEGs: Differentially expressed genes
GSCs: Glioblastoma stem cells
AML: Acute myeloid leukemia
PDAC: Pancreatic ductal adenocarcinoma
GBC: Advanced gallbladder carcinoma
